# Development of a nomogram based on the clinicopathological and CT features to predict the survival of primary pulmonary lymphoepithelial carcinoma patients

**DOI:** 10.1186/s12931-024-02767-5

**Published:** 2024-03-29

**Authors:** Kai Nie, Lin Zhu, Yuxuan Zhang, Yinan Chen, John Parrington, Hong Yu

**Affiliations:** 1 Department of Radiology, Shanghai Chest Hospital, Shanghai Jiao Tong University School of Medicine, No. 241 Huai-Hai West Road, Shanghai, 200030 P. R. China; 2 Department of Pharmacology, University of Oxford, Oxford, OX1 3QT UK

**Keywords:** Computed tomography, Lung cancer, Prognostic factors, Nomogram, Survival

## Abstract

**Background:**

The aim of this study was to develop a nomogram by combining chest computed tomography (CT) images and clinicopathological predictors to assess the survival outcomes of patients with primary pulmonary lymphoepithelial carcinoma (PLEC).

**Methods:**

113 patients with stage I–IV primary PLEC who underwent treatment were retrospectively reviewed. The Cox regression analysis was performed to determine the independent prognostic factors associated with patient’s disease-free survival (DFS) and cancer-specific survival (CSS). Based on results from multivariate Cox regression analysis, the nomograms were constructed with pre-treatment CT features and clinicopathological information, which were then assessed with respect to calibration, discrimination and clinical usefulness.

**Results:**

Multivariate Cox regression analysis revealed the independent prognostic factors for DFS were surgery resection and hilar and/or mediastinal lymphadenopathy, and that for CSS were age, smoking status, surgery resection, tumor site in lobe and necrosis. The concordance index (C‑index) of nomogram for DFS and CSS were 0.777 (95% CI: 0.703–0.851) and 0.904 (95% CI: 0.847–0.961), respectively. The results of the time‑dependent C‑index were internally validated using a bootstrap resampling method for DFS and CSS also showed that the nomograms had a better discriminative ability.

**Conclusions:**

We developed nomograms based on clinicopathological and CT factors showing a good performance in predicting individual DFS and CSS probability among primary PLEC patients. This prognostic tool may be valuable for clinicians to more accurately drive treatment decisions and individualized survival assessment.

**Supplementary Information:**

The online version contains supplementary material available at 10.1186/s12931-024-02767-5.

## Background

Primary pulmonary lymphoepithelial carcinoma (PLEC) is a unique and rare subtype of non-small-cell lung cancer (NSCLC), accounting for less than 0.7% of all NSCLCs [1–3]. PLEC was first reported in 1987 and histologically resembles undifferentiated nasopharyngeal carcinoma (NPC) [4]. From the epidemiological and etiological perspective, PLEC is more common in Asian ethnicities, tends to occur in relatively young and middle-aged individuals, and is generally considered to be closely related to Epstein–Barr virus (EBV) infection [5]. PLEC was previously classified as a subtype of large-cell lung cancer [6]. In 2015, the World Health Organization (WHO) classified it as one of the “other and unclassified carcinomas” [7]. In contrast, the latest 5th edition of the WHO classification of thoracic tumors in 2021 re-categorized PLEC as a subtype of squamous cell carcinomas (SCCs) [8]. The constantly changing classification of PLEC indicates the imperative need for further research.

Currently, the treatment of PLEC mainly follows the NCCN Clinical Practice Guidelines for NSCLC [9, 10]. Due to the rarity of primary PLEC, the standard of management for this disease is not still established [9]. In particular, mutations of commonly driven genes are lacking for PLEC patients, and targeted therapy drugs have little significance [11]. Therefore, the lack of treatment methods and experience for treating PLEC patients indicates an imperative need for individualized clinical management and precise survival prediction.

In a prognostic setting, the estimation of risk probability is rarely based on individual risk factors, as reliable estimates are insufficient. Discovering more prognostic factors and estimating based on multivariate models are now considered more reliable methods. Chest computed tomography (CT) is the routine imaging method for lung cancer detection and post-treatment management, and the CT image features have significant value in the diagnosis and prognosis of lung cancer [12, 13]. However, the relevant studies on imaging characteristics of primary PLEC are very few, and the cohort size of each published study was quite small [13–16]. Several reports have integrated clinical and pathological data from several PLEC patients for prognostic evaluation [17, 18], but the CT imaging features associated with the survival outcome of primary PLEC have not yet been described.

Therefore, this study aimed to develop a model including clinicopathological and CT features to estimate the disease-free survival (DFS) and cancer-specific survival (CSS) in patients with primary PLEC and to evaluate its clinical predictive ability and net benefit rate for individual survival estimation.

## Methods

This retrospective study was approved by the institutional review board (No. IS22019), and the requirement for written informed consent was waived.

### Patients

**Fig. 1:**
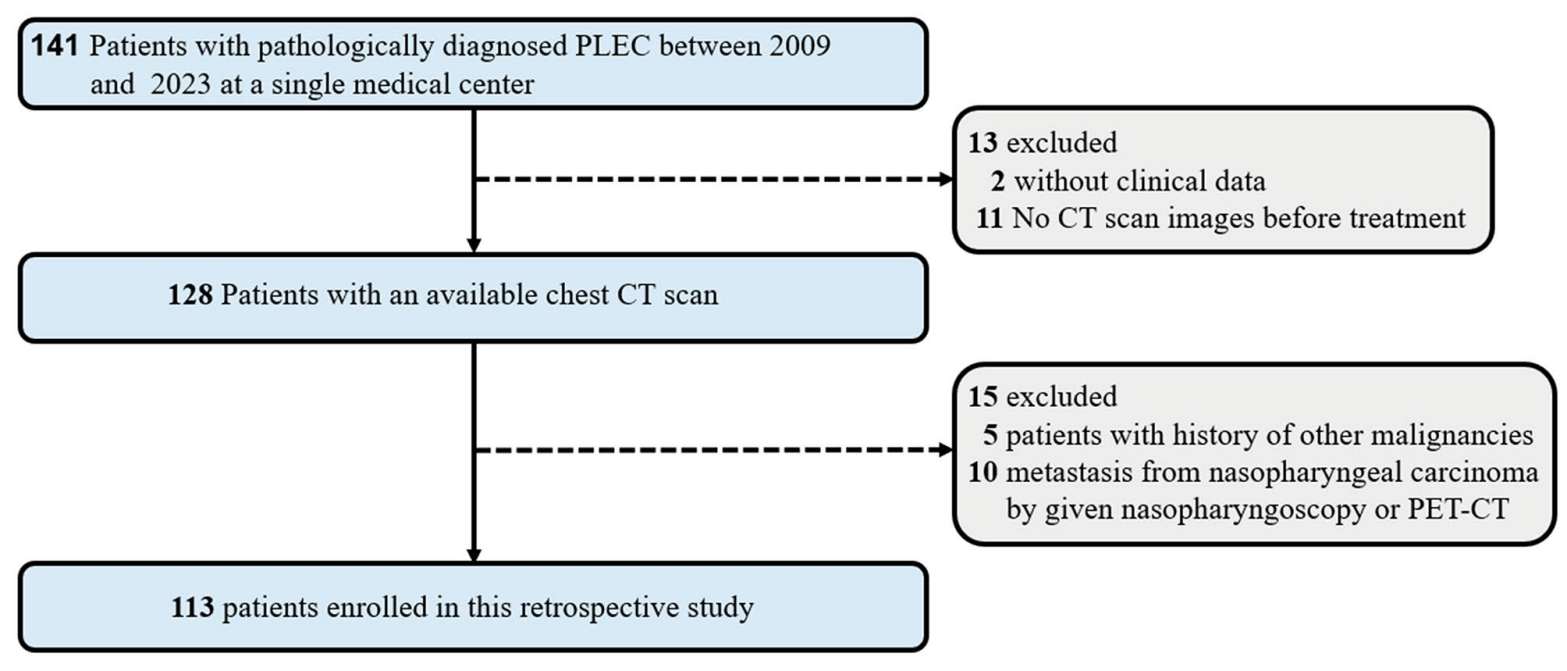
Flowchart shows patient selection

This study was conducted in patients with pathologically diagnosed PLEC between October 2009 and March 2023 at Shanghai Chest Hospital Affiliated to the Shanghai Jiao Tong University School of Medicine (Shanghai, China). In total, 141 cases were initially retrospectively recruited. The inclusion criteria were as follows: (1) CT scan was performed before treatment; (2) The diagnosis of primary PLEC was confirmed by fine-needle biopsy or complete surgical resection pathology; (3) The patient’s baseline characteristics and clinical data were complete. The exclusion criteria were as follows: (1) The past history of other malignancy, and (2) Metastasis of nasopharyngeal PLEC. Finally, A total of 113 patients were included in this study (51 males and 62 females; mean age, 56.8 years ± 11.5; range, 20–81 years). Figure 1 shows the patient recruitment pathway, along with the exclusion criteria. All primary PLEC tumors were reclassified based on the 5th edition of the WHO classification of Thoracic Tumors. Tumor staging was performed based on the American Joint Committee on Cancer TNM Staging Manual, 8th edition [19]. Among the 113 patients, 85 patients who underwent surgery provided pathological stage, while the remaining 28 patients who underwent non-surgical treatment provided clinical stage alone. We reviewed clinicopathological records and pre-treatment CT imaging data of all patients.

### Imaging examination protocol

Among the 113 patients, 34 patients underwent a plain chest CT, 79 cases underwent both unenhanced and enhanced CT. Somatom Definition AS (Siemens Medical Systems, Erlangen, Germany) and Brilliance 40 (Philips Medical Systems, the Netherlands Cleveland, state of Ohio, USA) scanners were used as the scanning machine. Patients were scanned at the end of inspiration during a single breath hold in the supine position. CT settings were as follows: tube voltage, 120 kVp; average tube current, 250 mA; pitch, 0.984; and section thickness, 1 mm. Scans covered the region from the top of the thoracic cage to the level of bilateral adrenal glands, and patients underwent a contrast-enhanced CT scan (non-ionic contrast medium, 60–80 mL). All imaging data were reconstructed using the standard algorithm and viewed with both lung window (window width, 1,500 HU; window level, − 500 HU) and mediastinal window (window width, 350 HU; window level, 50 HU).

### Image analysis

All post-processed images were interpreted retrospectively and independently by two experienced thoracic radiologists (HY and YNC) with 10 and 30 years of experience in chest imaging. The observers were blinded to the identities and clinical data of the patients. For all disagreements between the two observers on CT findings, the decisions were then reached by consensus. The location, shape, size, margin, interface, internal features, adjacent structures and CT attenuation values of the lesion were assessed. The definitions and scoring rules of morphological features are described in Table S1.

### Follow-up

CT, MRI, or PET/CT imaging was performed for the post-treatment disease status evaluation, and patients were evaluated once every six months within the first two years and then annually thereafter unless a specific clinical event emerged. The primary endpoint was DFS, which was defined from the date of initial histological diagnosis to the date of the first recorded evidence of clinical recurrence or distant metastasis as confirmed by histological evidence or death by any related causes. The secondary endpoint was CSS, calculated from the initial histological diagnosis to the date of death resulting from the progression of lung cancer (local and/or distant). The patient’s medical records and a telephone consultation were used for follow-up.

### Statistical analysis

Statistical analysis was performed using SPSS software, version 26.0 (SPSS Inc., Chicago, IL, USA), R software, version 3.0.1 (http://www.R-project.org) and X-tile software, version 3.6.1 (Yale University School of Medicine, New Haven, Conn). The nomogram, decision curve analysis curves and calibration curves were plotted by the *rms* package in R. Survival curve was plotted using Kaplan–Meier survival analysis and compared using the log-rank test with the *survminer* and *survival* package in R. Continuous variables are summarized as means and standard deviations if the distribution was normal or as medians and interquartile range (IQR) if the distribution was not normal. Categorical variables are reported as frequencies and percentages. Two-tailed *p* < 0.05 was considered statistically significant.

In this study, CT values were transformed into categorical variables and the optimal cut-off values were obtained by X-tile [20]. The repeatability for quantitative tumor size measurement was analyzed using the intraclass correlation coefficient (ICC). Reproducibility was defined as poor (ICC intraclass correlation coefficient < 0.75), moderate (ICC intraclass correlation coefficient = 0.75–0.90), or high (ICC intraclass correlation coefficient > 0.90) [21]. Interobserver agreement for qualitative variables of CT imaging was evaluated using Cohen’s kappa analysis. The κ value was interpreted as < 0.20, poor or slight agreement; 0.21–0.40, fair agreement; 0.41–0.60, moderate agreement; 0.61–0.80, good agreement; and 0.81–1.00, very good agreement [22].

Predictors for DFS and CSS were selected by Cox proportional hazards regression analysis. As PLEC is a rare tumor and the number of cases is not many and the events is less. In addition, CT findings of PLEC is a new insight and the prognostic analysis is exploring, therefore, those with a significant level of *p* ≤ 0.05 in univariate analysis and statistically insignificant but clinically significant were entered into the multivariate Cox regression method with a backward stepwise selection procedure. A nomogram with endpoints of 3- and 5-year CSS and DFS were constructed based on the multivariate Cox regression analysis results, respectively. Harrell’s concordance index (C-index) was measured to quantify the discriminative performance of nomograms. All internal validations were performed using a bootstrapping method with 500 resamples. The calibration curves of nomogram were then drawn for the 3-year and 5-year CSS and DFS of the patients, which illustrated both survival probabilities predicted by nomogram and the observed probabilities. The decision curve analysis was conducted to estimate the clinical usefulness of the nomogram by quantifying the net benefits at different threshold probabilities. Finally, subjects were divided into high- and low-risk groups according to the median on the nomogram scores obtained from the constructed model. The Kaplan–Meier method and log-rank test were applied to calculate and compare risk group differences. Data between groups were compared using the independent t-test. Furthermore, categorical variables were presented with count (%) and were compared using the χ^2^ test.

## Results

### Patients baseline characteristics

**Fig. 2:**
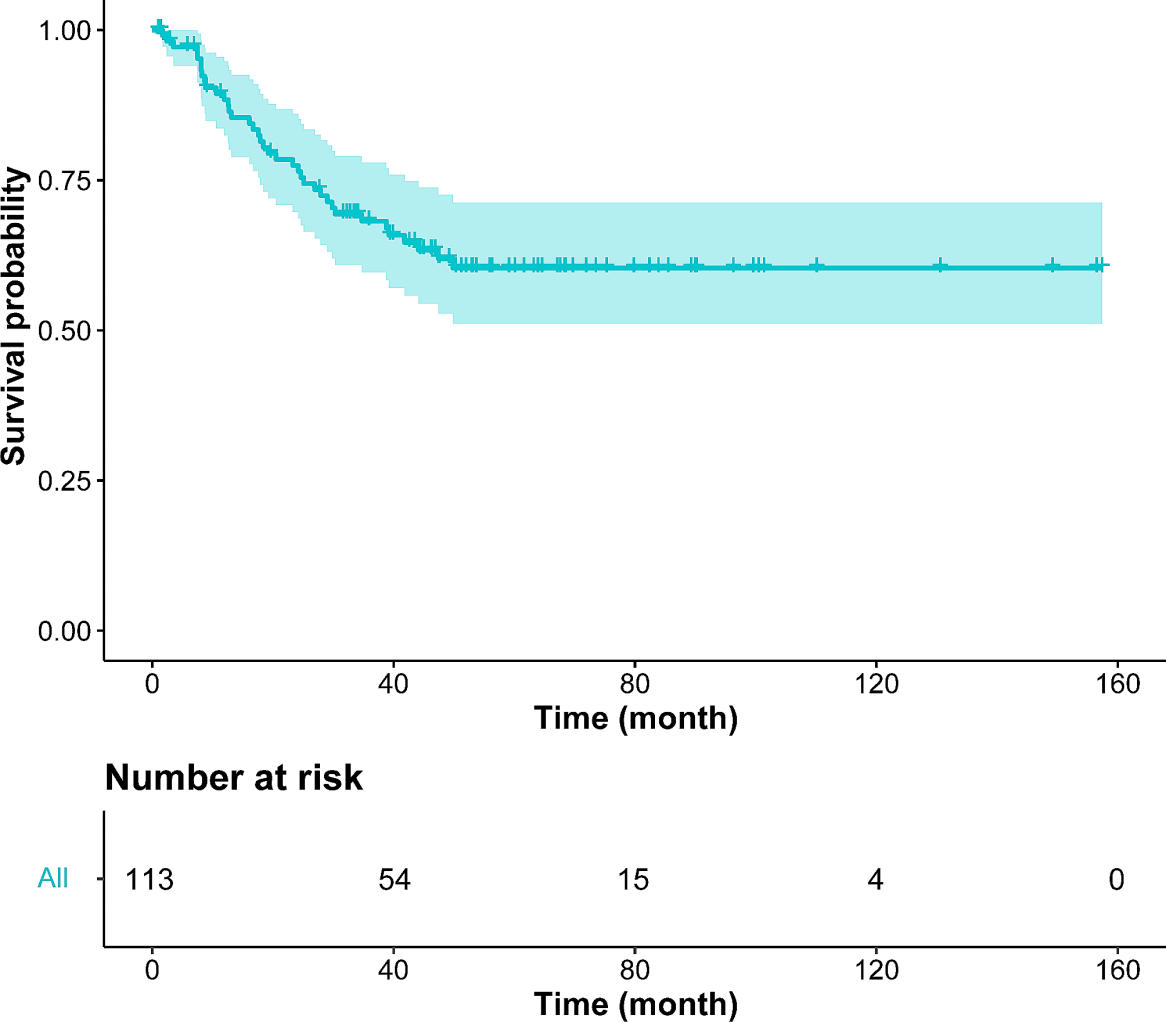
KaplanMeier curve for DFS of total patients

The clinicopathological features of all PLEC patients are shown in Table 1. In the primary PLEC cohort, the median follow-up time was 53.1 months (range: 1–157.4 months). The DFS and CSS of all PLEC patients are shown in Fig. 2 and Fig S1a. The median DFS and CSS was not reached. The 1-, 3-, and 5-year CSS rates were 99.0, 88.6 and 76.1%, respectively. The 1-, 3-, and 5-year DFS rates were 88.4, 68.2, and 60.4%, respectively. The optimal cut-off value for CT attenuation was 37.8 HU which was obtained by X-tile. The ICC for the quantitative measurement of tumor size was 0.997 (95% confidence interval [CI]: 0.995–0.998; *P* < 0.001). The interobserver reproducibility for qualitative CT imaging features was good or excellent (κ, 0.73–1.00). Table S2 showed a detailed description of the inter–reader agreement. The detailed CT features of the 113 patients are summarized in Table 2.

**Table 1:** Baseline demographics and clinicopathological characteristics

Variables	All patients (*N* = 113)	
	Category	Total (%)
Age	Mean ± SD, years	56.8 ± 11.5
	< 60	67 (59.3)
	≥ 60	46 (40.7)
Sex		
	Male	51 (45.1)
	Female	62 (54.9)
Smoking status		
	Never smoker	98 (86.7)
	Ever/current smoker	15 (13.3)
Main complaint		
	Cough with or without blood-tinged sputum	29 (25.7)
	Hemoptysis	4 (3.5)
	Chest pain	4 (3.5)
	Asymptomatic	76 (67.3)
AJCC 8th stage		
	I	43 (38.1)
	II	19 (16.8)
	III	36 (31.9)
	IV	15 (13.3)
T stage		
	T1	27 (23.9)
	T2	53 (46.9)
	T3	16 (14.2)
	T4	17 (15.0)
N stage		
	N0	62 (54.9)
	N1	7 (6.2)
	N2	26 (23.0)
	N3	18 (15.9)
M stage		
	M0	98 (86.7)
	M1	15 (13.3)
CYFRA21-1		
	Normal	71 (62.8)
	Elevated	42 (37.2)
EGFR (*n* = 57) #		
	Mutated	0 (0)
	Wild	57 (100)
ALK (*n* = 45) #		
	Mutated	0 (0)
	Wild	45 (100)
KRAS (*n* = 40) #		
	Mutated	0 (0)
	Wild	40 (100)
Treatment procedure		
	Surgery alone	48 (42.5)
	Surgery and adjuvant therapy*	37 (32.7)
	Other therapy†	28 (24.8)
EBER in situ hybridization (*N* = 83)#		
	Negative	3 (3.6)
	Positive	80 (96.4)
Recurrence		
	Local	22 (19.5)
	Regional	8 (7.1)
	Distant metastasis	8 (7.1)
Death		
	Cancer specific deaths	20 (17.7)
	Treatment related mortality	1 (0.9)

Data are numbers of patients, with percentages in parentheses

*SD*, Standard deviation; *CYFRA21-1*, Cytokeratin fragment antigen 21 − 1; *EBER*, EBV-encoded small non-polyadenylated RNAs; *EGFR*, Epidermal growth factor receptor; *ALK*, Anaplastic lymphoma kinase; *KRAS*, Kirsten rat sarcoma viral oncogene

* Adjuvant therapy, including chemotherapy and radiotherapy

† Other therapy, including chemotherapy, immunotherapy and radiotherapy

# Denotes missing data for some patients

**Table 2:** CT morphological features of all PLEC patients

CT Imaging Features	All patients (*N* = 113)	
	Category	Total (%)
Maximum diameter	median (IQR), cm	3.4 (2–4.7)
CT attenuation value	mean ± SD, HU	37.3 ± 10.5
Tumor localization		
	Central	25 (22.1)
	Peripheral	88 (77.9)
Morphology		
	Irregularity	9 (8.0)
	Round/oval	104 (92.0)
Tumor site in lobe		
	Right lung	64 (56.6)
	RUL	13 (11.5)
	RML	20 (17.7)
	RLL	21 (18.6)
	Involved multiple lobes	10 (8.8)
	Left lung	49 (43.4)
	LUL	20 (17.7)
	LLL	27 (23.9)
	Involved multiple lobes	2 (1.8)
Interface		
	Ill-defined	2 (1.8)
	Well-defined, smooth	69 (61.1)
	Well-defined, coarse	42 (37.2)
Margin		
	Lobulation	92 (81.4)
	Spiculation	20 (17.7)
	Spine-like process	19 (16.8)
Internal characteristics		
	CT bronchograms	
	None	17 (15.0)
	Dilatation, distortion	17 (15.0)
	Cut-off	79 (70.0)
	Calcification	10 (8.8)
	Necrosis	19 (16.8)
Adjacent structure		
	Vascular convergence	67 (59.3)
	Vascular encasement	57 (50.4)
	Hilar and/or mediastinal LAP	54 (47.8)
	Pleural and/or pericardial effusion	9 (8.0)

Data are numbers of patients, with percentages in parentheses

*SD*, standard deviation; *IQR*, interquartile ranges; *RUL*, right upper lobe; *RML*, right middle lobe; *RLL*, right lower lobe; *LUL*, left upper lobe; *LLL*, left lower lobe; *LAP*, lymphadenopathy; *CT*, computed tomography

### Developing a clinicopathological and CT imaging-based nomogram to predict DFS and CSS

**Fig. 3:**
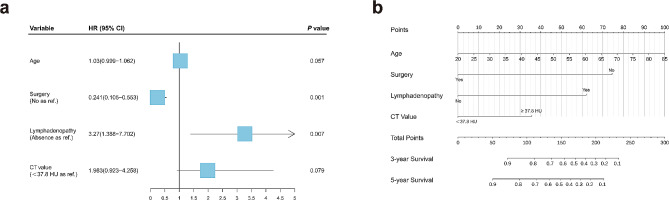
The forest plot of factors obtained through multivariate COX regression analysis for DFS **(a)**; The nomogram established for prediction of DFS **(b)**

The results of the univariate and multivariate Cox analysis for predictive factors are presented in Table 3 and Table S3. According to multivariate analysis results for DFS, a total of four variables were retained through backward stepwise selection; only the surgery resection (*p* = 0.001, HR = 0.24; 95% CI 0.11–0.55) and Hilar and/or mediastinal LAP (*p* = 0.007, HR = 3.27; 95% CI 1.39–7.70) being significant independent prognostic factors. According to multivariate analysis results for CSS, a total of six variables were retained, and the following variables showed significantly independent prognostic factors: age (*p* < 0.001, HR = 1.13; 95% CI 1.06–1.20), smoking status (*p* = 0.038, HR = 4.15; 95% CI 1.09–15.88), surgery resection (*p* < 0.001, HR = 0.05; 95% CI 0.01–0.19), tumor site in lobe (*p* = 0.014, HR = 0.29; 95% CI 0.11–0.78), hilar and/or mediastinal LAP (*p* = 0.038, HR = 4.49; 95% CI 1.09–18.53) and necrosis (*p* = 0.011, HR = 3.96; 95% CI 1.37–11.50). The HRs and 95% CIs for the multivariate Cox regression analysis for remaining DFS and CSS risk factors are shown as forest plots in Fig. 3a and Fig. S1b. Consequently, the nomograms for predicting the probability of 3-and 5-year DFS and CSS of all primary PLECs were developed using the risk factors combined with clinical and CT Imaging features (Fig. 3b and Fig. S1c). To use the nomogram, a vertical line needs to be delineated to the point raw to assign point values for each factor, and the total points are calculated as the sum of the risk points of all risk factors.

**Table 3:** The results of univariate and multivariate analysis of disease-free survival

Characteristics	Univariate		Multivariate	
	HR (95% CI)	*P*-value	HR (95% CI)	*P*-value
Clinical factor				
Age (per 1-year increase)	1.00 (0.97–1.03)	0.993	1.03 (1.00-1.06)	0.057
Gender (Male as ref.)	1.70 (0.87–3.33)	0.120	…	…
Smoking status (Never as ref.)	1.88 (0.86–4.11)	0.112	…	…
Symptom (Absence as ref.)	3.20 (1.69–6.07)	<0.001	…	…
CYFRA21-1 (Normal as ref.)	4.14 (2.14–8.04)	<0.001	…	…
Surgery resection (No as ref.)	0.15 (0.08–0.29)	<0.001	0.24 (0.11–0.55)	**0.001**
Pathologic factor				
T stage (T1/2 as ref.)	2.42 (1.27–4.62)	0.007	…	…
M stage (M0 as ref.)	5.14 (2.37–11.13)	<0.001	…	…
Chest CT factor				
Morphology (Round/oval as ref.)	3.81 (1.33–10.94)	0.013	…	…
Tumor site in lobe (Left as ref.)	0.64 (0.34–1.22)	0.176	…	…
Hilar and/or mediastinal LAP (Absence as ref.)	5.55 (2.61–11.78)	<0.001	3.27 (1.39–7.70)	**0.007**
Vascular encasement (Absence as ref.)	1.34 (0.71–2.55)	0.365	…	…
Necrosis (Absence as ref.)	1.89 (0.90-4.00)	0.095	…	…
Pleural and/or pericardial effusion (Absence as ref.)	3.90 (1.51–10.06)	0.005	…	…
CT value (<37.8 HU as ref.)	3.14 (1.48–6.64)	0.003	1.98 (0.92–4.26)	0.079

*LAP*, lymphadenopathy; *CYFRA21-1*, Cytokeratin fragment antigen 21 − 1; *HR*, Hazard ratio; *CI* Confidence interval; *CT*, Computed tomography

### The discrimination, net benefit and predictive capacity of the nomogram

**Fig. 4:**
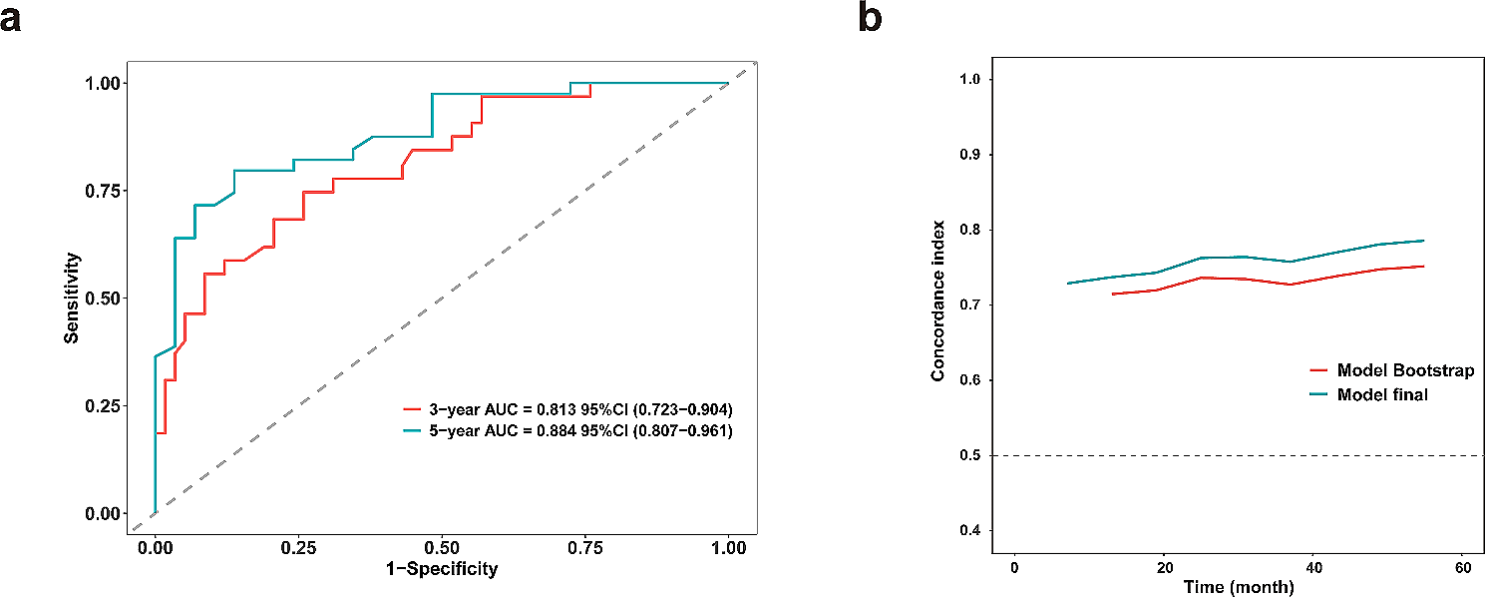
Area under the curves at 3-year and 5-year were calculated to assess the prognostic accuracy for DFS **(a)**; Timedependent Cindex of nomogram of all PLEC patient (blue lines) and internally validated using a bootstrap resampling method (red lines) for DFS **(b)**

**Fig. 5:**
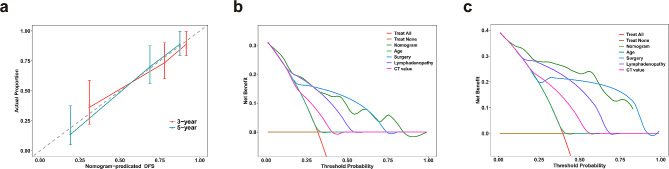
Calibration curves for 3, 5year DFS **(a)** of nomogram predictions; Decision curve analysis of nomogram for 3year DFS **(b)** and 5year DFS **(c)** of PLEC patients. The red line is the net benefit of a strategy of treating all people; the brown line is the net benefit of treating no people. The yaxis indicates the overall net benefit, which is calculated by summing the benefits (true-positive results) and subtracting the harms (false-positive results), weighting the latter by a factor related to the relative harm of undetected cancer compared with the harm of unnecessary treatment

The C-indexes of the nomograms for DFS and CSS prediction in the dataset were 0.777 (95% CI: 0.703–0.851) and 0.904 (95% CI: 0.847–0.961), respectively. The performance of nomogram for clinical prediction was evaluated using the area under the receiver-operating characteristic (ROC) curve (AUC) (Fig. 4a and Fig. S1d), the 3- and 5-year AUC for DFS were 0.820 and 0.901, respectively, and those for CSS were 0.941 and 0.922, respectively. Moreover, time-dependent C-index analysis also showed that the nomograms exhibited good prognostic accuracy in clinical outcome prediction for DFS or CSS. A similar result was also observed in internal validation using a bootstrap resampling method (red lines) (Fig. 4b and Fig. S1e). The calibration plots of the prognostic nomograms in predicting 3- and 5-year DFS and CSS demonstrated good coincidences between the estimated risk and observed risk (Fig. 5a and Fig. S1f). The decision curve analysis for 3-and 5-year DFS and CSS showed that the combined nomogram had a higher overall net benefit than each clinical and CT imaging factor across the majority of the range of reasonable threshold probabilities (Fig. 5b,c and Fig. S1g,h).

### Risk stratification for PLEC patients

**Fig. 6:**
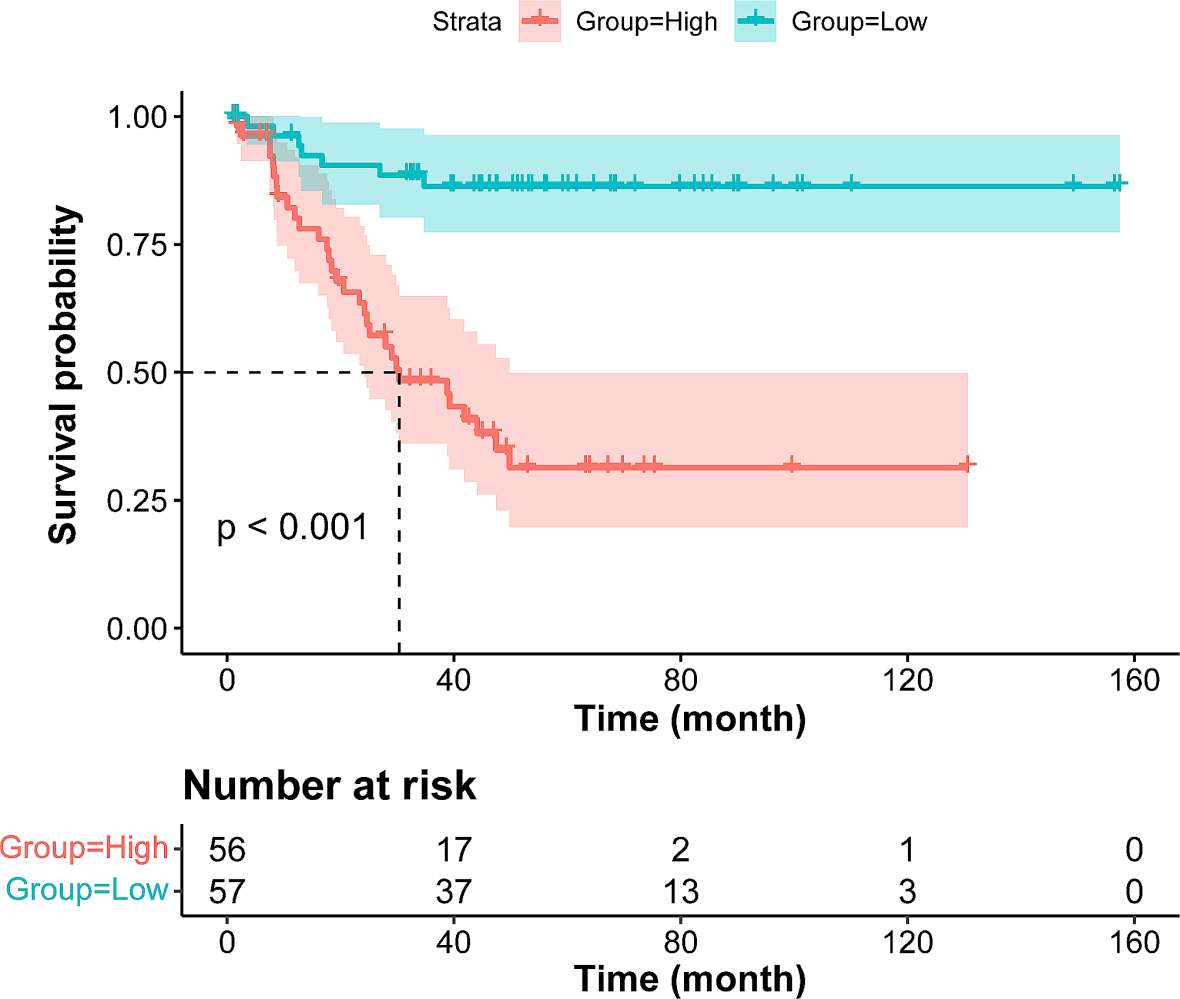
KaplanMeier curve for DFS based on the nomogram prediction

To assess whether the primary PLEC patients could be effectively separated into two proposed risk groups based on the nomograms, we calculated each patient’s total point and used the median to determine the optimal cut-off value. Patients with nomogram scores less than or equal to the median were classified as low-risk groups, and those with scores greater than the median were classified as high-risk groups. According to the range of total points, the Kaplan- Meier curves highlighted the appropriateness of distinguishing the patients’ survival for DFS and CSS in all the subgroups. The groups were obtained considering the total point distribution of our cohort. Compared with the high-risk group (red lines), group low-risk (blue lines) represent patients with better prognoses (Fig. 6 and Fig. S1i). In order to explore individual factor comparisons within the clinical, pathologic, and chest CT factors between the high-risk and low-risk groups, we conducted a statistical comparison of various risk factors for patients with different risks, and the relevant results are shown in Table S4, 5.

## Discussions

In our cohort, it was found that female patients and non-smokers accounted for the majority. Most patients were found during physical examinations, while a few had symptoms such as cough with or without blood-tinged sputum, similar to other NSCLCs without specificity [23, 24]. No common mutation-driving genes in lung cancer were observed in our study. The above characteristics are consistent with the results reported in previous studies [2, 3, 18]. In addition, we restaged 113 PLEC patients in this cohort according to the 8th edition of the TNM staging system. The results showed that nearly half of the PLEC patients have a higher TNM stage (III + IV, 45.2%) at initial diagnosis, indicating that surgical resection is no longer feasible for treatment and requires multidisciplinary collaborative treatment.

Routine initial and follow-up examinations of lung lesions mainly rely on CT scans in clinical practice. Therefore, we evaluated the morphological CT manifestations of 113 patients with primary PLEC before treatment. The results showed that the median maximum diameter on CT imaging was 3.4 cm (IQR, 2–4.7 cm), and the average CT value on plain CT scan was 37.3 ± 10.5 HU. This indicates that PLEC often presents as large, soft tissue-dense masses on CT. Tumors are mostly located in the right lobe of the lung and are more common in peripheral types. However, few studies have reported that PELC mainly manifested as the central type of lung cancer [14, 15]; it may be related to the small number of included cases.

Further CT imaging analysis showed that most PLECs exhibit solitary, well-defined solid nodules or masses, with lobulation sign more common, spiculation sign less common, and bronchogram cut-off more common. These characteristics are consistent with previous research results [14, 15, 25]. Moreover, the hilar and/or mediastinal LAP was more common in this cohort (54/113, 47.8%), indicating that primary PLEC is prone to lymph node metastasis. With a summary of these CT scanning characteristics, we attempted to integrate the clinical, pathological, and CT imaging features of all PLEC patients in the cohort. We conducted long-term follow-ups to discover more potential indicators for predicting survival risk.

Based on univariate and multivariate analysis for DFS and CSS, PLEC patients who did not receive surgery had a worse CSS and DFS because patients who have not undergone surgery are often in the advanced stage of TNM staging. On the multivariate analysis, hilar and/or mediastinal LAP was an independent prognostic factor for DFS and CSS. Previous studies also reported that nodal stage in the TNM system and lymph node involvement were independent prognostic factors for post-operative recurrence-free survival (RFS) in stage I-IIIa PLEC patients [26]. Our findings suggest that as a non-invasive examination method, pre-treatment hilar and/or mediastinal LAP on CT images in stage I-IV PLEC patients can provide an independent value for predicting survival outcomes. On the multivariate analysis for CSS, we found that age, smoking history, tumor site in the lobe, and necrosis signs were independent prognostic factors. Older PLEC patients and those with a history of smoking have a higher risk of death. A previous study found that PLEC patients with lesions in the left lobe of the lung seemed to have a poorer DFS in univariate analysis (*p* = 0.051), but they only included 30 cases of PELC [17]. Our study expanded the size of the study cohort and covariates, further demonstrating that the location of tumors in PLEC patients was an independent prognostic factor for CSS, indicating that patients with tumors in the left lung lobe have a higher risk of survival. This may be due to the lack or difficulty in 4 L lymph node dissection during routine surgical resection in patients with left lung cancer, resulting in a poorer prognosis compared to right lung cancer patients [27]. It is worth noting that female lung cancer patients often have better prognosis than males [28], while in the univariate analysis of this study, the prognosis of females was worse than that of males. We consider this may be due to the small number of included cases. In particular, age and smoking history were not significant in univariate analysis in this cohort (*p* = 0.148, 0.546, respectively) but became independent prognostic factors for CSS when included in multivariate analysis. This fully indicates that age and smoking history, once combined with other prognostic factors, have an impact on the prognosis of PLEC patients.

Furthermore, patients with necrosis on CT images had poorer CSS; this might because necrosis often occurs in large tumors with insufficient blood supply, while larger tumors have higher T staging and poorer prognosis. These conclusions may help clinicians understand the relationship between CT findings and patient survival in PLEC patients. In addition, we also found that symptomatic patients with elevated CYFRA21-1, irregularity shape on CT images, CT values (<37.8 HU as ref.) and patients with pleural and/or pericardial effusion had worse prognosis for both DFS and CSS on univariate analysis (*p*<0.05), but not significant on multivariate analysis. This suggests that these variables may potentially correlate with the prognosis of PLEC patients. Especially in this cohort, up to 37.2% of PLEC patients had elevated levels of CYFRA21-1, which had been proven to be highly expressed in SCCs [29], indirectly demonstrating the necessity for primary PLEC to be classified as a subtype of lung SCCs.

Based on the Cox multivariate regression analysis results, we developed nomograms model that included multiple clinical and CT imaging prognostic factors to predict DFS and CSS in PLEC patients. Our nomograms showed the C-indexes of the overall dataset were higher than 0.7 and AUCs greater than 0.8 under the 3-year and 5-year ROC curves, indicating that the nomograms have an excellent discrimination performance for predicting clinical outcomes. The results of time-dependent C-index analysis further showed that this combined nomogram still had good predictive ability after undergoing 500 resamples of internal bootstrap validation. Moreover, the 3-year and 5-year decision curves and calibration plots for CSS and DFS showed that the nomograms we developed had strong prediction accuracy and overall net benefits and could evaluate clinical relevance without additional validation data in traditional decision analysis methods [30]. In addition, this nomogram can successfully classify PLEC patients into high and low-risk subgroups. Compared to the low-risk group, the high-risk group had the worst prognosis (*p* < 0.001). In summary, our nomogram, which combines pre-treatment CT imaging and clinicopathological features, has great potential in clinical application for predicting the prognosis of PLEC patients and may assist clinicians in the decision-making process, allowing patients to obtain more benefits.

However, our research still has some limitations. Firstly, our research findings are based on a retrospective design; therefore, this study cannot exclude all potential inherent biases. Secondly, our data were obtained from a single cancer center, and the sample size was relatively small, the prediction model of prognosis was sufficient for DFS but for CSS. Finally, we did not find enough samples for external validation.

## Conclusions

In conclusion, we first studied the relationship between CT imaging features and the prognosis of primary PLEC patients, and the identified CT imaging features may serve as biomarkers for prognostic risk stratification in PLEC patients. At the same time, we have developed new nomograms that combine clinicopathological and CT imaging features for individualized survival risk assessment of primary PLEC patients. Before conducting multicenter studies with larger samples in future, these nomograms were developed for simple usage and readily available prognostic tools may have potential value in promoting treatment decision-making and individualized prognosis prediction more effectively in clinical practice.

### Electronic supplementary material

Below is the link to the electronic supplementary material.

Supplementary Material 1. Fig. S1 Kaplan Meier curve for CSS of total patients (a), the forest plot of factors obtained through multivariate COX regression analysis for CSS (b), the nomogram established for prediction of CSS (c), area under the curves at 3-year and 5-year were calculated to assess the prognostic accuracy for CSS (d), time dependent C index of nomogram of all PLEC patient (blue lines) and internally validated using a bootstrap resampling method (red lines) for CSS (e), calibration curves for 3 , 5 year CSS of nomogram predictions (f), decision curve analysis of nomogram for 3 year CSS (g) and 5 year CSS (h) of PLEC patients and Kaplan Meier curve for CSS based on the nomogram prediction (i).

Supplementary Material 2

## Data Availability

Any reasonable requests for access to available data underlying the results reported in this article will be considered. Such proposals should be submitted to the corresponding author.

## Abbreviations

CT: Computed tomography
PLEC: Pulmonary lymphoepithelial carcinoma
DFS: Disease-free survival
CSS: Cancer-specific survival
C‑index: Concordance index
CYFRA21-1: Cytokeratin fragment antigen 21 − 1
EBER: EBV-encoded small non-polyadenylated RNAs
EGFR: Epidermal growth factor receptor
ALK: Anaplastic lymphoma kinase
KRAS: Kirsten rat sarcoma viral oncogene
SD: Standard deviation
IQR: Interquartile ranges
RUL: Right upper lobe
RML: Right middle lobe
RLL: Right lower lobe
LUL: Left upper lobe
LLL: Left lower lobe
LAP: Lymphadenopathy
HR: Hazard ratio
CI: Confidence interval
AUC: Area under the curve
