# Supplementary figures and images for: Development of a nomogram based on the clinicopathological and CT features to predict the survival of primary pulmonary lymphoepithelial carcinoma patients
Source: Respir Res. 2024 Mar 29;25:144. doi: 10.1186/s12931-024-02767-5 (PMC10981313; doi:10.1186/s12931-024-02767-5)

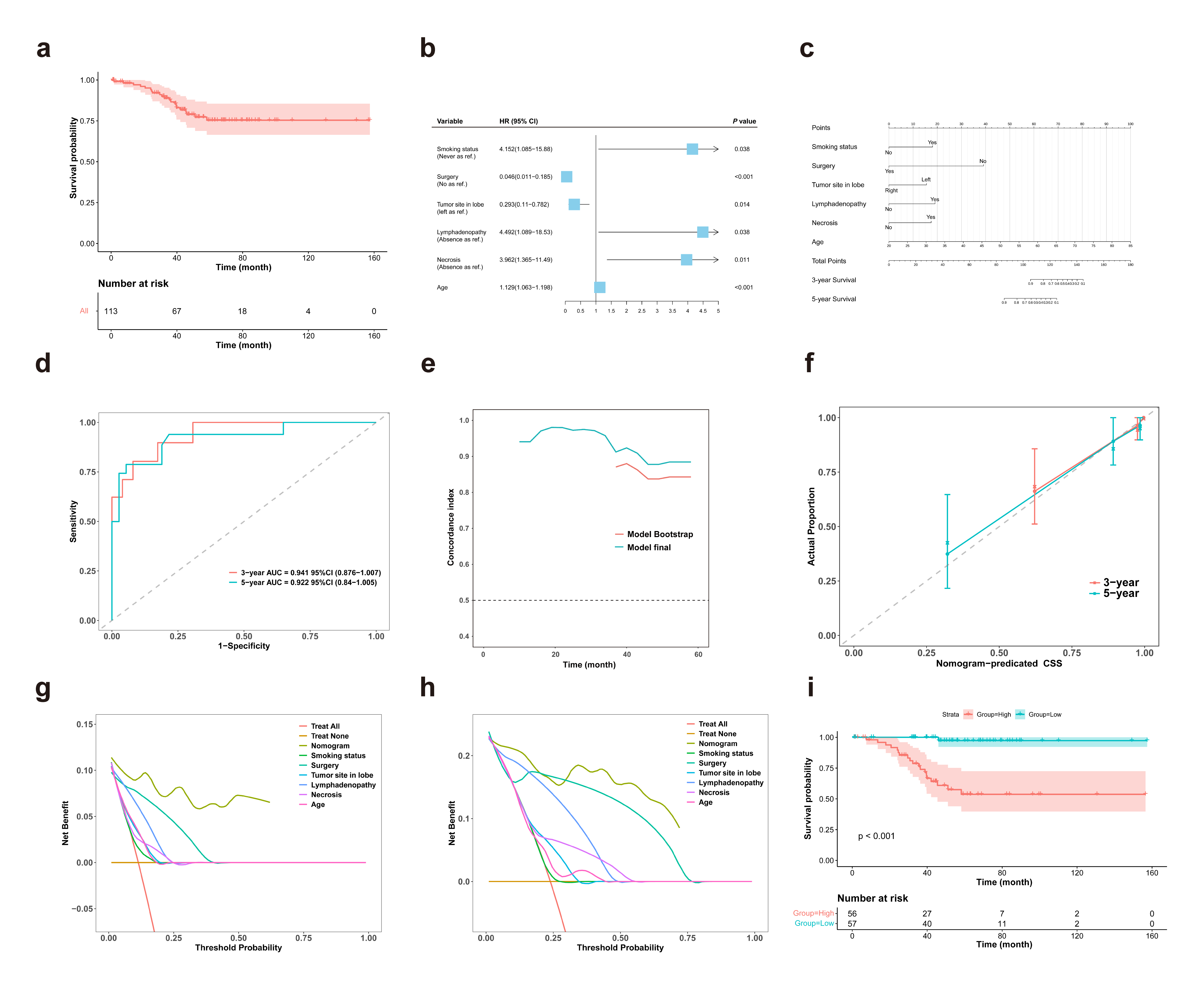

Supplement: Supplementary file 1 — Supplementary Material 1. Fig. S1 Kaplan Meier curve for CSS of total patients (a), the forest plot of factors obtained through multivariate COX regression analysis for CSS (b), the nomogram established for prediction of CSS (c), area under the curves at 3-year and 5-year were calculated to assess the prognostic accuracy for CSS (d), time dependent C index of nomogram of all PLEC patient (blue lines) and internally validated using a bootstrap resampling method (red lines) for CSS (e), calibration curves for 3 , 5 year CSS of nomogram predictions (f), decision curve analysis of nomogram for 3 year CSS (g) and 5 year CSS (h) of PLEC patients and Kaplan Meier curve for CSS based on the nomogram prediction (i). [file 12931_2024_2767_MOESM1_ESM.tif]
