# Supplementary material for: Development of a nomogram based on the clinicopathological and CT features to predict the survival of primary pulmonary lymphoepithelial carcinoma patients
Source: Respir Res. 2024 Mar 29;25:144. doi: 10.1186/s12931-024-02767-5 (PMC10981313; doi:10.1186/s12931-024-02767-5)
Supplement: Supplementary file 2 — Supplementary Material 2 [file 12931_2024_2767_MOESM2_ESM.docx]

Table S1. The definitions and scoring rules of morphological features

| Feature | Definition | Scoring |
| --- | --- | --- |
| Maximum diameter | Longest diameter of the maximum cross section | Unit, centimeter |
| Tumor localization | Central, tumor originated from the segmental or more proximal bronchi; peripheral, tumor originated from the subsegmental bronchi or more distal airway | 1, central; 2, peripheral |
| Morphology | Shape of tumor on the multiplanar reconstructed (MPR) images | 1, irregular; 2, round/oval |
| Interface | Interface of the tumor-lung | 1, ill-defined; 2, well-defined and smooth; 3, well-defined but coarse |
| Marginal features |  |  |
| Lobulation | The surface of the tumor that showed a wavy or scalloped configuration | 0, no; 1, yes |
| Spiculation | Short lines radiating from the margin of the tumor | 0, no; 1, yes |
| Spine-like process | Thick protuberance between lobulation and spiculation on the surface of the tumor, which can be dendritic, finger-like or blunt triangular. | 0, no; 1, yes |
| Internal characteristics |  |  |
| CT bronchograms | Air - containing bronchus within the tumor | 1, none; 2, dilated/distorted;3, cut-off |
| Calcification | The high-density shadows within the tumor often show CT values greater than 100 HU on the mediastinal window | 0, no; 1, yes |
| Necrosis | Low density shadows without enhancement found inside the tumor | 0, no; 1, yes |
| Adjacent structure |  |  |
| Vascular convergence | Convergence of vessels to the tumor | 0, no; 1, yes |
| Vascular encasement | Tumor mass involving the vascular encasement |  |
| Lymphadenopathy | Enlarged lymph nodes (hilar or mediastinal) with short-axis diameter greater than 1 cm | 0, no; 1, yes |
| Pleural and/or pericardial effusion | Mediastinal window reveals fluid density in pleural cavity or pericardium | 0, no; 1, yes |

Table S2. Interobserver agreement of CT imaging features in PLEC

| Parameter | Cohen’s kappa coefficient (95% CI) | *P* value |
| --- | --- | --- |
| Tumor location (left or right) | 1.00 (1.00-1.00) | <0.001 |
| Shape (yuan or buguize) | 0.809 (0.599-1.019) | <0.001 |
| Interface | 0.914(0.828-1.000) | <0.001 |
| Lobulation | 0.732(0.588-0.877) | <0.001 |
| Spiculation | 0.903(0.796-1.011) | <0.001 |
| Spine-like process | 0.832(0.689-0.974) | <0.001 |
| CT bronchograms | 0.804(0.704-0.903) | <0.001 |
| Calcification | 1.00 (1.00-1.00) | <0.001 |
| Necrosis | 0.857(0.735-0.978) | <0.001 |
| Vascular convergence | 0.893(0.810-0.976) | <0.001 |
| Vascular encasement | 0.929(0.861-0.997) | <0.001 |
| Hilar and/or mediastinal LAP | 0.965(0.916-1.013) | <0.001 |
| Pleural and/or pericardial effusion | 0.890(0.741-1.040) | <0.001 |

**Table S3** The results of univariate and multivariate analysis of cancer specific survival

| **Characteristics** | **Univariate** | | **Multivariate** | |
| --- | --- | --- | --- | --- |
|  | **HR (95% CI)** | ***P*-value** | **HR (95% CI)** | ***P*-value** |
| Clinical factor |  |  |  |  |
| Age (per 1-year increase) | 1.03 (0.99-1.07) | 0.135 | 1.13 (1.06-1.20) | **＜0.001** |
| Gender (Male as ref.) | 3.73 (1.25-11.17) | 0.019 |  |  |
| Smoking status (Never as ref.) | 1.48 (0.49-4.43) | 0.484 | 4.15 (1.09-15.88) | **0.038** |
| Symptom (Absence as ref.) | 3.77 (1.54-9.21) | 0.004 | … | … |
| CYFRA21-1 (Normal as ref.) | 5.29 (2.03-19.79) | 0.001 | … | … |
| Surgery resection (No as ref.) | 0.10 (0.04-0.25) | ＜0.001 | 0.05 (0.01-0.19) | **＜0.001** |
| Pathologic factor |  |  |  |  |
| T stage (T1/2 as ref.) | 3.06 (1.27-7.36) | 0.013 | … | … |
| M stage (M0 as ref.) | 8.81 (3.31-23.49) | ＜0.001 | … | … |
| Chest CT factor |  |  |  |  |
| Morphology (Round/oval as ref.) | 9.37 (2.95-29.72) | ＜0.001 | … | … |
| Tumor site in lobe (Left as ref.) | 0.36 (0.14-0.90) | 0.028 | 0.29 (0.11-0.78) | **0.014** |
| Hilar and/or mediastinal LAP (Absence as ref.) | 9.39 (2.74-32.18) | ＜0.001 | 4.49 (1.09-18.53) | **0.038** |
| Vascular encasement (Absence as ref.) | 3.90 (1.42-10.75) | 0.008 | … | … |
| Necrosis (Absence as ref.) | 3.90 (1.59-9.56) | 0.003 | 3.96 (1.37-11.50) | **0.011** |
| Pleural and/or pericardial effusion (Absence as ref.) | 7.44 (2.68-20.71) | ＜0.001 | … | … |
| CT value (＜37.8 HU as ref.) | 2.60 (0.94-7.17) | 0.065 | … | … |

*LAP*, lymphadenopathy; *CYFRA21-1*, cytokeratin fragment antigen 21-1; *HR,* hazard ratio; *CI*  confidence interval; *CT*, computed tomography

**Table S4** A comparison of the characteristics between the High- and Low-risk groups for disease-free survival.

| **Variables** | **High-risk Group**  **(n = 56)** | **Low-risk Group (n = 57)** | **Statistics**  **(t/χ**^2^**)** | ***P*-value** |
| --- | --- | --- | --- | --- |
| Clinical factor |  |  |  |  |
| Age |  |  |  |  |
| Mean ± SD, years | 58.8 ± 12.6 | 54.9 ± 10.0 | t = 1.781 | 0.078 |
| Gender |  |  |  |  |
| Male | 20 | 31 | χ^2^ = 3.977 | 0.059 |
| Female | 36 | 26 |  |  |
| Smoking status |  |  |  |  |
| Never | 50 | 48 | χ^2^ = 0.632 | 0.427 |
| Yes | 6 | 9 |  |  |
| Symptom |  |  |  |  |
| No | 28 | 48 | χ^2^ = 15.012 | ＜0.001 |
| Yes | 28 | 9 |  |  |
| CYFRA21-1  Normal | 22 | 49 | χ^2^ = 26.356 | ＜0.001 |
| Elevated | 34 | 8 |  |  |
| Surgery resection  No | 28 | 0 | χ^2^ = 37.888 | ＜0.001 |
| Yes | 28 | 57 |  |  |
| Pathologic factor |  |  |  |  |
| T stage  T1/2 | 30 | 50 | χ^2^ = 15.932 | ＜0.001 |
| T3/4 | 26 | 7 |  |  |
| N stage |  |  |  |  |
| N0 | 15 | 47 | χ^2^ = 35.353 | ＜0.001 |
| N1/2/3 | 41 | 10 |  |  |
| M stage |  |  |  |  |
| M0 | 41 | 57 | χ^2^ = 17.605 | ＜0.001 |
| M1 | 15 | 0 |  |  |
| Chest CT factor |  |  |  |  |
| Morphology |  |  |  |  |
| Round/oval | 49 | 55 | χ^2^ = 2.009 | 0.156 |
| Irregular | 7 | 2 |  |  |
| Tumor site in lobe |  |  |  |  |
| Left | 24 | 25 | χ^2^ = 0.012 | 0.914 |
| Right | 32 | 32 |  |  |
| Hilar and/or mediastinal LAP |  |  |  |  |
| Absence | 8 | 52 | χ^2^ = 67.150 | ＜0.001 |
| Presence | 48 | 5 |  |  |
| Vascular encasement |  |  |  |  |
| Absence | 18 | 38 | χ^2^ = 13.468 | ＜0.001 |
| Presence | 38 | 19 |  |  |
| Necrosis |  |  |  |  |
| Absence | 40 | 54 | χ^2^ = 10.972 | 0.001 |
| Presence | 16 | 3 |  |  |
| Pleural and/or pericardial effusion |  |  |  |  |
| Absence | 47 | 57 | χ^2^ = 7.882 | 0.005 |
| Presence | 9 | 0 |  |  |
| CT value |  |  |  |  |
| ＜37.8 HU | 15 | 35 | χ^2^ = 13.722 | ＜0.001 |
| ≥37.8 HU | 41 | 22 |  |  |

*LAP*, lymphadenopathy; *CYFRA21-1*, Cytokeratin fragment antigen 21-1; *HR*, Hazard ratio; *CI*  Confidence interval; *CT*, Computed tomography

**Table S5** A comparison of the characteristics between the High- and Low-risk groups for cancer-specific survival.

| **Variables** | **High-risk Group**  **(n = 56)** | **Low-risk Group (n = 57)** | **Statistics**  **(t/χ**^2^**)** | ***P*-value** |
| --- | --- | --- | --- | --- |
| Clinical factor |  |  |  |  |
| Age |  |  |  |  |
| Mean ± SD, years | 60.1 ± 11.8 | 53.6 ± 10.3 | t = 3.140 | 0.002 |
| Gender |  |  |  |  |
| Male | 21 | 30 | χ^2^ = 2.612 | 0.106 |
| Female | 35 | 27 |  |  |
| Smoking status |  |  |  |  |
| Never | 46 | 52 | χ^2^ = 2.025 | 0.155 |
| Yes | 10 | 5 |  |  |
| Symptom |  |  |  |  |
| No | 29 | 47 | χ^2^ = 12.066 | 0.001 |
| Yes | 27 | 10 |  |  |
| CYFRA21-1  Normal | 26 | 45 | χ^2^ = 12.791 | ＜0.001 |
| Elevated | 30 | 12 |  |  |
| Surgery resection  No | 28 | 0 | χ^2^ = 37.888 | ＜0.001 |
| Yes | 28 | 57 |  |  |
| Pathologic factor |  |  |  |  |
| T stage  T1/2 | 33 | 47 | χ^2^ = 7.563 | 0.006 |
| T3/4 | 23 | 10 |  |  |
| N stage |  |  |  |  |
| N0 | 17 | 45 | χ^2^ = 26.933 | ＜0.001 |
| N1/2/3 | 39 | 12 |  |  |
| M stage |  |  |  |  |
| M0 | 41 | 57 | χ^2^ = 17.605 | ＜0.001 |
| M1 | 15 | 0 |  |  |
| Chest CT factor |  |  |  |  |
| Morphology |  |  |  |  |
| Round/oval | 49 | 55 | χ^2^ = 2.009 | 0.156 |
| Irregular | 7 | 2 |  |  |
| Tumor site in lobe |  |  |  |  |
| Left | 28 | 21 | χ^2^ = 1.991 | 0.158 |
| Right | 28 | 36 |  |  |
| Hilar and/or mediastinal LAP |  |  |  |  |
| Absence | 14 | 46 | χ^2^ = 35.193 | ＜0.001 |
| Presence | 42 | 11 |  |  |
| Vascular encasement |  |  |  |  |
| Absence | 20 | 36 | χ^2^ = 8.511 | 0.004 |
| Presence | 36 | 21 |  |  |
| Necrosis |  |  |  |  |
| Absence | 40 | 54 | χ^2^ = 10.972 | 0.001 |
| Presence | 16 | 3 |  |  |
| Pleural and/or pericardial effusion |  |  |  |  |
| Absence | 48 | 56 | χ^2^ = 4.463 | 0.035 |
| Presence | 8 | 1 |  |  |
| CT value |  |  |  |  |
| ＜37.8 HU | 21 | 29 | χ^2^ = 2.049 | 0.152 |
| ≥37.8 HU | 35 | 28 |  |  |

*LAP*, lymphadenopathy; *CYFRA21-1*, Cytokeratin fragment antigen 21-1; *HR*, Hazard ratio; *CI*  Confidence interval; *CT*, Computed tomography
